# Systematic review of patient-reported outcome item libraries in cancer research: an EORTC Quality of Life Group study

**DOI:** 10.1093/jnci/djag039

**Published:** 2026-02-16

**Authors:** Rosemary Peacock, Christopher Bedding, Bonnie Pacheco, Hayat Hamzeh, Claire Piccinin, Alexandra Gilbert

**Affiliations:** Leeds Institute for Medical Research, University of Leeds, St James’s Hospital, Leeds, United Kingdom; Leeds Institute for Medical Research, University of Leeds, St James’s Hospital, Leeds, United Kingdom; Quality of Life Department, European Organisation for Research and Treatment of Cancer (EORTC) Headquarters, Brussels, Belgium; Leeds Institute for Medical Research, University of Leeds, St James’s Hospital, Leeds, United Kingdom; Quality of Life Department, European Organisation for Research and Treatment of Cancer (EORTC) Headquarters, Brussels, Belgium; Leeds Institute for Medical Research, University of Leeds, St James’s Hospital, Leeds, United Kingdom

## Abstract

**Background:**

Patient-reported outcome (PRO) item libraries support flexible PRO assessment in cancer research by facilitating the development of customized item lists. However, little is known about the use of item lists in clinical research. This systematic review addresses this by assessing utilization of PRO item libraries and lists in oncology research.

**Methods:**

A systematic review of MEDLINE, Embase, and CINAHL identified cancer studies using PRO item libraries to develop item lists, regardless of study design, published between October 2021 and September 2025. Key features of item library usage were extracted and analyzed descriptively.

**Results:**

A total of 78 studies were included (25 trials and feasibility, 53 observational). The Patient-Reported Outcomes version of the Common Terminology Criteria for Adverse Events system was frequently used (49 of 78), and symptom assessment was the most common application (63 of 78). Item lists were implemented across different settings including novel treatments (19 of 78) and rare cancers (15 of 78). Most item lists were derived from a single PRO item library (72 of 78). In some studies, there was additional customization such as item wording changes (5 of 78) or addition of items adapted from the item library (9 of 78). Item selection methods included literature (32 of 78), patient involvement (8 of 78) and consultation with health-care professionals (11 of 78). Many studies (40 of 78) did not report methods used.

**Conclusions:**

PRO item libraries are increasingly used to create customized item lists in oncology research, primarily for symptom assessment. However, reporting practices for methods used are inconsistent, highlighting the need for standardized guidelines for reporting PRO item lists in clinical trials and routine care to improve transparency, reproducibility, and quality.

## Introduction

Patient-reported outcome (PRO) measures play a key role in assessing the impact of cancer and its therapies from a patient-centered viewpoint. They are implemented within clinical research settings, including clinical trials and observational studies, and increasingly as part of clinical care.^1-5^ A wide selection of valid, reliable, and fit-for-purpose measures have been developed using rigorous methods to assess patient perspectives of health-related quality of life (HRQOL).^6^ These measures cover diverse disease and treatment types and include a broad range of health-related concepts spanning disease- and treatment-related symptoms and functional issuessuch as role and physical functioning.^7^

The development of a PRO measure is a lengthy undertaking involving several stages and high numbers of participants to ensure validity.^8^^,^^9^ To preserve validity, such measures are often static and offer limited flexibility regarding the content and administration. However, there is a growing need for more flexiblity when assessing PROs within cancer research. PRO item libraries offer a novel and important approach to PRO assessment by facilitating the creation of customized questionnaires, referred to as item lists.^10^ Item lists are created from preexisting questions (ie, items) from within an item library and can provide greater validity than ad hoc self-developed items by offering a standardized approach and a useful supplement to standard validated PRO measures.^11^

Item libraries have been made increasingly accessible by the main PRO measurement systems in oncology. Four PRO item libraries are dedicated to patient reporting of impacts and effects of cancer and its treatment.^12-16^ The European Organisation for Research and Treatment of Cancer (EORTC) item library,^12^ Functional Assessment of Chronic Illness Therapy (FACIT) Searchable Library,^13^ and MD Anderson Symptom Inventory (MDASI) symptom library^14^ are populated by PRO items used in their respective validated questionnaires, which can be used to create item lists that supplement the standard measures. Developed as a companion to the Common Terminology Criteria for Adverse Events (CTCAE),^17^ the National Cancer Institute PRO version of the Common Terminology Criteria for Adverse Events Measurement System (PRO-CTCAE) Item library^15^^,^^16^ covers symptomatic adverse events for PRO assessment in cancer clinical trials, with versions for adult and pediatric populations. The Patient-Reported Outcomes Measurement Information System (PROMIS)^18^ is not cancer specific and is a computer adaptive testing tool, whereby PRO items are organized into item banks designed to support computer adaptive testing and short-form approaches to questionnaire design and administration.

Areas where greater measurement flexibility is thought to be beneficial include assessing the impact of novel treatments, which may require inclusion of additional items capturing symptomatic adverse events or functional impacts (eg, not included within existing PRO measures); in rare cancers where the population is not large enough to enable psychometric development of a new questionnaire;^10^ and in early-phase trials assessing patient-reported tolerability.^19^ Other areas include the assessment of symptomatic adverse events in clinical research^20^^,^^21^ and clinical monitoring of disease- and treatment-associated symptoms and function in clinical care settings.^22-24^

Increased recognition by regulators, such as the US Food and Drug Administration^11^^,^^25^ and the European Medicines Agency,^26^ of the role of PROs as important endpoints in cancer clinical trials for the assessment of tolerability through measurement of, for example, symptomatic adverse events and HRQOL, are contributing to a growing demand for measures. Moreover, the need to support flexible measurement of PROs through the use of item libraries has been highlighted as an important approach to ensure that PRO measures cover relevant toxicities and remain tailored to the given context of use.^26^

An international working group^10^ examining guidance on the use of item libraries identified a need for internationally agreed recommendations to support their use for the creation of item lists in cancer clinical trials. These recommendations covered 9 areas: methods used to drive item selection, use of single items or multi-item scales, psychometric properties, bias, unexpected issues, ordering lists, recall periods, minimization of patient burden, and use with PRO measures. Furthermore, they identified that little was published in terms of guidance on how item libraries should be used within cancer research. This review aims to establish how PRO item libraries (and the item lists derived from them) are currently implemented in cancer clinical trials and observational research, including clinical care settings. The findings from this review will support the development of international consensus-driven guidelines for item library use.

## Methods

### Search strategy

Searches were conducted initially between October 1, 2021, and October 1, 2023, and then updated between October 1, 2023, and September 18, 2025, via MEDLINE, Embase, and CINAHL. To facilitate the identification of publications that did not directly use the term “PRO item library” in the title or abstract, a broad 2-concept search was developed. The 2 concepts were “cancer” and “PRO item library,” and for both, key terms and subject headings were combined using Boolean logic “AND” and “OR” in the searches. Examples of key terms were “supplementary” and “additional” to capture the possibility of items being added to a questionnaire or measure. Searches were developed in MEDLINE and adapted for the other databases (see supplementary materials).

### Study selection

References and data were managed using Covidence systematic review software^27^ and Microsoft Excel. Identified references were exported into Covidence, with duplicates subsequently removed. The literature screening process was documented using the Preferred Reporting Items for Systematic reviews and Meta-Analyses reporting guidelines.^28^ The screening process was conducted in 2 stages: title and abstract screening followed by full-text screening. A team of 5 reviewers (R.P., C.B., B.P., C.P., H.H.) participated in the screening process. The records were independently screened by 2 reviewers to minimize bias and ensure consistency. Discrepancies between reviewers were resolved through discussion with a sixth reviewer (A.G.).

We identified studies (regardless of study design) that used a cancer PRO item library to develop PRO item lists. We defined “PRO item library” as a database of questions (items) or multi-item scales that measure HRQOL domains, including disease-related symptoms, symptomatic adverse events, functioning, and overall health status.^10^ A “PRO item list” was defined as a customized list of PRO items selected from an item library.^10^ We included studies involving patients or survivors of all ages with any cancer type. We did not exclude studies that recruited subpopulations with other medical conditions or diseases. Publications were restricted to a 4-year period (October 1, 2021, to September 18, 2025) for pragmatic reasons to limit the number of articles retrieved arising from taking a broad search strategy as described above. Studies on module or questionnaire development, linguistic validation, or other forms of validation were excluded. Studies using computer adaptive testing questionnaires, computer adaptive testing short forms, or computer adaptive testing item banks (eg, PROMIS) were outside the scope of the review and not included.

Inclusion criteria are as follows:

1. All cancer patients and survivors, any age, any cancer stage with any type of treatment for cancer
2. Studies with mixed cancer types or other diseases as well as cancer
3. Studies using an item library to select single items, multi-items, scales from any of the major PRO measurement systems (PRO-CTCAE Measurement System, EORTC item library, MDASI Symptom Library, FACIT Searchable Library, and PROMIS)
4. Any stage clinical trial or other research study design
5. Quantitative or qualitative research
6. Published in English language
7. Research on humans
8. Nonpharmacological studies
9. Published between October 1, 2021 and September 18, 2025

Exclusion criteria are as follows:

1. Measure was not a PRO or from one of the main PRO measurement systems (PRO-CTCAE Measurement System, EORTC item library, MDASI Symptom Library, FACIT Searchable Library, and PROMIS)
2. Methodological or development studies, including validation, linguistic validation, module or questionnaire development, or computer adaptive test questionnaires
3. Studies for which full text was not available
4. Reviews

### Quality assessment

The Mixed Methods Assessment Tool^29^ is recommended as a critical appraisal tool for systematic reviews of studies using mixed methods (eg, randomized and nonrandomized studies)^30^ and was completed by a team of 4 reviewers (R.P., C.B., H.H., C.P.). Variations in assessment were discussed with a fifth reviewer (A.G.) to achieve consensus. Publications were not excluded on the basis of the quality assessment. Data are reported in supplementary materials.

### Data extraction, synthesis, and analysis

Data were independently extracted by 2 reviewers (R.P., C.B., B.P., H.H.) for each study. Differences between reviewers were resolved through discussion to reach consensus. Data were synthesized descriptively to determine which item libraries were used, for what purposes, in which contexts, and the methods employed to develop item lists. Studies were categorized into 2 groups: trials and feasibility studies or observational studies. The study population was classified into study participants with cancer diagnosed by disease site, and where studies also included participants with other medical conditions or diseases was noted. The intervention was classified according to the treatment studied and grouped into broad categories. Study PRO objectives were classified as (1) assessment of efficacy, effectiveness, or clinical benefit (often including PROs measuring disease- and treatment-related symptoms and the impact of cancer on a function or aspect of everyday life); or (2) reporting of symptomatic adverse events (including toxicities or tolerability); or (3) other as defined in the study methods. Classifications were not mutually exclusive. Classifications were discussed by the review team (R.P., C.B., B.P., H.H., C.P., A.G.) to reach consensus.

The study context in which item lists were implemented was categorized as (1) use in an early-phase trial, (2) for rare diseases, (3) for novel treatments, (4) for clinical monitoring, and (5) other settings that were noted. Classifications were not mutually exclusive. Methods used to select item lists were identified and categorized as (1) using literature sources, (2) consulting health-care professionals, and (3) involving patients or patient representatives. The use of free-text options that allow patients to record issues not included in the PRO measures were noted, as was whether respondent burden had been addressed in the methods. Data categories and definitions are reported in supplementary materials. The systematic review protocol is published on PROSPERO (Registration number: CRD42023468774).^31^

## Results

### Study characteristics

**Figure 1.**
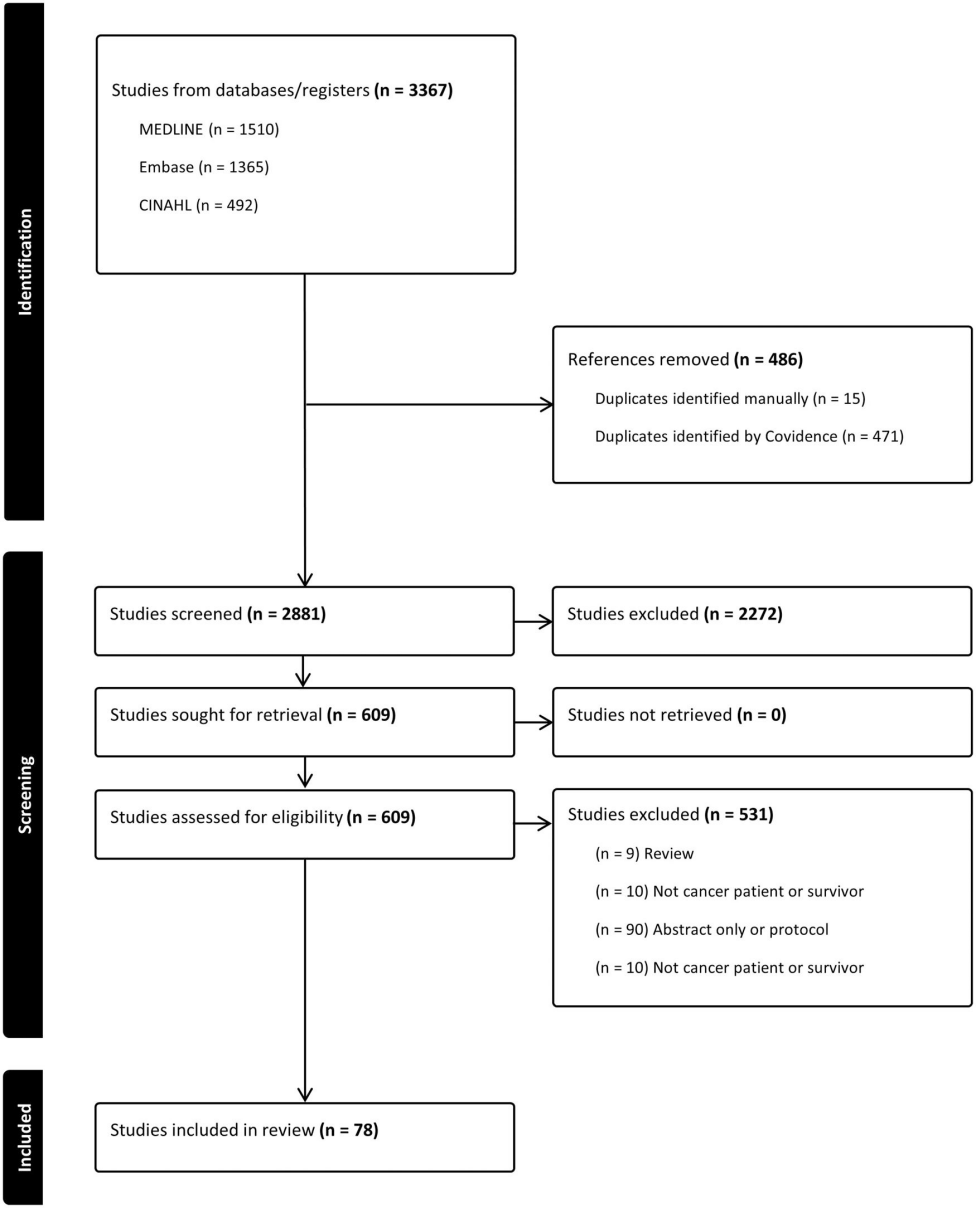
Preferred Reporting Items for Systematic reviews and Meta-Analyses (PRISMA) flowchart.

We identified 3367 references from the electronic databases (Figure 1: Preferred Reporting Items for Systematic reviews and Meta-Analyses (PRISMA) flowchart); 486 duplicates were removed, 2881 titles and abstracts were screened, 609 full texts were assessed, and after applying inclusion and exclusion criteria, 78 articles were included. In the initial review conducted between October 1, 2021, and October 1, 2023, a total of 33 articles were included, compared with 45 articles over the later 2-year period (from October 1, 2023, to September 18, 2025), demonstrating the increased usage of item libraries over time. Table 1 summarizes the included studies. A total of 25 studies were classified as trials and feasibility studies and 53 as observational studies. Table 2 summarizes the study characteristics by study design. In the quality assessment, studies were further categorized as quantitative randomized control trial (14 of 78), quantitative nonrandomized control trial (24 of 78), quantitative descriptive (33 of 78) or mixed methods design (6 of 78), or qualitative (1 of 78). Further details can be seen in supplementary materials.

**Table 1.** Study characteristics.

Author (year)	Study design category: trial and feasibility or observational (Country)	Cancer population (participants with)	Intervention or treatment type	PRO objective^a^	Validated PROs measures	Item library and other customizations	Item No.	Names of issues (No. of items per issue)
Bandos (2025)^32^	Trial and feasibility(United States)	Breast	Various cancer treatments	Symptomatic adverse event tracking	N/A	PRO-CTCAE	23	Fatigue (2); mucositis (2); diarrhea (1); numbness and tingling (2); rash (1); itching (1); cough (2); shortness of breath (2); abdominal pain (3); joint pain (3); nausea (2); decreased appetite (2)
Basch et al. (2023)^33^	Trial and feasibility(United States)	Gastrointestinal	Chemoradiation	Tracking symptomatic adverse events	Kettering bowel function instrument; EQ-5D	PRO-CTCAE;Other customization: Prostate Health-Related QOL questionnaire: 1 item - bladder function; International Prostate Symptom Score: 1 item - bladder function; International Index of Erectile Function: 1 item (male patients only); Female Sexual Function Index: 1 item	28	Anxiety (3); appetite loss (2); constipation (1); depression (3); diarrhea (1); dysphagia (1); dyspnea (1); edema (3); fatigue (2); mucositis (2); nausea (2); neuropathy (2); pain (3); vomiting (2)
Carter (2025)^34^	Trial and feasibility(United States)	Gynecological	Surgery	Other: HRQOL	FACT cervix; Female Sexual Function Index; Gynecologic Cancer Lymphedema Questionnaire; Impact of Event Scale; Reproductive Concerns Scale	PROMIS Other customization: 6 novel items	8	PROMIS: relationship status (1); current sexual activity (1)
Chung et al. (2022)^35^	Trial and feasibility(United States)	Gastrointestinal	Palliative care	Efficacy, effectiveness or clinical benefit; tracking symptomatic adverse events	FACT-G; Psychological distress thermometer	PRO-CTCAE	45	Not specified: 45 items relevant to pancreatic cancer selected by research team
Compton (2025)^36^	Trial and feasibility(United States)	Gastrointestinal	Chemotherapy	Symptomatic adverse event tracking	N/A	PRO-CTCAE	2	Peripheral neuropathy (2)
Culakova et al. (2022)^37^	Trial and feasibility(United States)	Various cancers	Chemotherapy	Efficacy, effectiveness or clinical benefit; tracking symptomatic adverse events	N/A	PRO-CTCAE	24	Fatigue (1); pain (1); anorexia (1); insomnia (1); shortness of breath (1); constipation (1); dry mouth (1); diarrhea (1); memory (1); concentration (1); numbness and tingling (1); taste (1); arm/leg swelling (1); headaches (1); nausea (1); dizziness (1); blurry vision (1); ringing in ears (1); difficulty swallowing (1); hair loss (1); vomiting (1); mouth and throat sores (1); hand foot syndrome (1); cracking at corners of mouth (1)
de Almeida (2023)^38^	Trial and feasibility(United States)	Prostate	Various cancer treatments	Symptomatic adverse event tracking	N/A	PRO-CTCAE	14	Abdominal pain (3); diarrhea (1); fatigue (2); anorexia (2); nausea (2); vomiting (2); rash (1); pruritus (1)
Fridriksdottir (2023)^39^	Trial and feasibility (Iceland)	Various cancers	Chemotherapy	Efficacy, effectiveness or clinical benefit	Edmonton Symptom Assessment System-Revised; Distress Thermometer and Problem	MDASI Other customization: 3 items added; source not specified	9	MDASI: symptom interference with walking (1); activity (1); working (including housework) (1); relations with other people (1); enjoyment of life (1); mood (1) Source not specified: overall quality of life (1); family support (1); perceived quality of care (1)
Gomaa (2023)^40^	Trial and feasibility (United States)	Gastrointestinal	Chemotherapy	Symptomatic adverse event tracking; efficacy/effectiveness/clinical benefit	FACT-G; Memorial Symptom Assessment scale	PRO-CTCAE	35	Fever (2); pain (3); fatigue (2); bruising (1); shortness of breath (2); cough (2); palpitations (2); diarrhea (1); nausea (2); vomiting (2); decreased appetite (3); constipation (1); bloating (2); change of taste (1); loss of bowel control (3); tingling (2); dizziness (2); insomnia (1)
Hummel (2025)^41^	Trial and feasibility (United States)	Lung	Immunotherapy	Symptomatic adverse event tracking; efficacy, effectiveness or clinical benefit	QLQ-C30; QLQ-LC13; Patient Global Impression of Severity and Change	FACIT; PRO-CTCAE	17	Arm or leg swelling (3); heart palpitations (2); headache (3); anxiety (3); shivering or shaking chills (2); rash (1); problems with concentration (2); GP5 symptom burden (1)
Hungria (2025)^42^	Trial/feasibility (United States)	Multiple myeloma	Immunotherapy/targeted therapy	Symptomatic adverse event tracking Other: HRQOL	QLQ-C30; EQ-5D; VAS	EORTC; FACIT; PRO-CTCAE	39	Abdomen pain (3); blurred vision (2); constipation (1); cough (2); decreased appetite (2); fatigue (2); itchy (1); loose or watery stool (1); mouth or throat sores (2); nausea (2); nosebleeds (2); numb or tingling hands and feet (2); pain or burning urination (1); problems tasting food or drink (1); shivering or shaking chills (2); shortness of breath (2); vomiting (2); watery eyes (2); bone pain; back pain; hip pain; pain in arm or shoulder; chest pain; pain increased with activity; GP5 symptom burden (1)
Kaveenuntachai (2025)^43^	Trial and feasibility (Thailand)	Breast	Chemotherapy	Symptomatic adverse event tracking; efficacy, effectiveness or clinical benefit	QLQ-C30; QLQ-BR23	PRO-CTCAE	24	Number of items not specified: dry mouth; mouth/throat sores; taste changes; decreased appetite; nausea; vomiting; constipation; diarrhea; shortness of breath; rash; hair loss; nail discoloration; sensitivity to sunlight; skin darkening; dizziness; general pain; headache; pain and swelling at the injection site; insomnia; fatigue; anxiety; discouragement; sadness; missed expected menstrual periods; vaginal dryness; hot flashes; fever
Knoerl et al. (2022)^44^	Trial and feasibility (United States)	Various cancers	Chemotherapy	Tracking symptomatic adverse events Other: clinical support algorithm development	N/A	PRO-CTCAE; EORTC Other customization: self-developed items	20	PRO-CTCAE: numbness and tingling (2); pain (1); EORTC: motor and sensory subscales (17)
Kurosawa (2024)^45^	Trial and feasibility (Japan)	Various cancers	Chemotherapy	Symptomatic adverse event tracking	N/A	PRO-CTCAE	28	Dry mouth (1); mouth/throat sore (1); taste changes (1); decreased appetite (1); nausea (1); vomiting (1); bloating (1); constipation (1); diarrhea (1); abdominal pain (1); shortness of breath (1); cough (1); swelling (1); heart palpitations (1); skin dryness (1); itching (1); numbness and tingling (1); blurred vision (1); concentration (1); memory problems (1); pain (1); insomnia (1); fatigue (1); anxiety (1); discouragement (1); sadness (1); painful urination (1); urinary frequency (1)
Lv (2025)^46^	Trial and feasibility (China)	Lung	Surgery	Efficacy, effectiveness or clinical benefit	MDASI-LC	EORTC	4	Fever (1); shortness of breath (1); pain (1); coughing blood/cough (1)
Ma et al. (2023)^47^	Trial and feasibility (United States)	Various cancers	Chemoradiation	Tracking symptomatic adverse events	PROMIS (specific tool used not reported)	Customization of PRO-CTCAE: included more attributes (frequency, severity and/or interference) than specified in PRO-CTCAE	Not specified	Core items specific to radiation: radiation skin reaction; skin darkening; general pain; fatigue Items specific to each disease Only provided detail of items for lower gastrointestinal: decreased appetite, nausea, vomiting, gas, bloating, constipation, diarrhea, abdominal pain, fecal incontinence, frequent urination, and painful urination
Madariaga et al. (2022)^48^	Trial and feasibility (Canada)	Gynecological	Chemotherapy and targeted therapy	Tracking symptomatic adverse events	N/A	PRO-CTCAE	18	Abdominal pain (3); anxiety (3); bloating (2); diarrhea (1); difficulty swallowing (1); fatigue (2); mucositis (2); nausea (2); vomiting (2)
Martin et al. (2022)^49^	Trial and feasibility (United States)	Blood cancers	Immunotherapy	Other: HRQOL	QLQ-C30; EQ-5D	EORTC	4	Feeling restless or agitated (1); thinking about their illness (1); being worried about dying (1); worried about future health (1)
Nelson et al. (2023)^50^	Trial and feasibility (United States)	Gastrointestinal	Chemoradiation	Tracking symptomatic adverse events	N/A	PRO-CTCAE	29	Taste change (1); decreased appetite (2); nausea (2); vomiting (2); gas (1); loose or watery stools (1); abdominal pain (3); fecal incontinence (2); skin burn from radiation (1); pain (3); vaginal discharge (1); vaginal dryness (1); painful urination (1); urinary urgency (2); urinary frequency (2); urine color change (1); loss of urine control (2); self-report options (1)
Nielsen et al. (2022)^51^	Trial and feasibility (Denmark)	Various cancers	Chemotherapy	Efficacy, effectiveness or clinical benefit; tracking symptomatic adverse events	North Central Cancer Treatment Group PRO- chemotherapy-induced peripheral neuropathy (specific measures not reported)	PRO-CTCAE Other customization: 4 items added	3	General pain (3)
Ohri (2025)^52^	Trial and feasibility (United States)	Lung	Chemoradiation	Symptomatic adverse event tracking	N/A	PRO-CTCAE	12	Dysphagia (1); anorexia (1); nausea (1); dyspnea (1); fatigue (1); cough (1); wheezing (1); dermatitis (1); dizziness (1); anxiety (1); anhedonia (1); depression (1)
Oliveira (2024)^53^	Trial and feasibility (United States)	Breast	Hormone therapy	Symptomatic adverse event tracking; efficacy, effectiveness or clinical benefit	QLQ-C30; QLQ-BR23; Patient Global Impression of Treatment Tolerability; EQ-5D-5L; Patient Global Impression of Severity/Change	PRO-CTCAE	7	Mouth/throat sores (2); loose/watery stools (1); rash (1); itchy skin (1); numbness or tingling in hands or feet (2)
Rugo (2024)^54^	Trial/feasibility (United States)	Breast	Targeted therapy	Symptomatic adverse event tracking	QLQ-C30; EQ-5D-5L	PRO-CTCAE	16	Decreased appetite (2); nausea (2); vomiting (2); constipation (1); diarrhea (1); abdominal pain (3); shortness of breath (2); hair loss (1); fatigue (2)
Saito (2025)^55^	Trial and feasibility (Japan)	Breast	Chemotherapy	Symptomatic adverse event tracking	N/A	PRO-CTCAE	18	Nausea (2); vomiting (2); anorexia (2); taste changes (1); dry mouth (1); hiccups (2); constipation (1); diarrhea (1); dizziness (2); concentration (2); insomnia (2)
Yeung et al. (2022)^56^	Trial and feasibility (United States)	Gynecological	Radiotherapy	Efficacy, effectiveness or clinical benefit; Tracking symptomatic adverse events	EQ-5D; FACT cervix	PRO-CTCAE Other customization: items from other questionnaire	5	Diarrhea (1); abdominal pain (2); bowel control (2)
Adesoye et al. (2023)^57^	Observational (United States)	Breast	Chemotherapy	Tracking symptomatic adverse events	N/A	Customization of PRO-CTCAE: included more attributes (frequency, severity and/or interference) than specified in PRO-CTCAE	27	Arm/leg swelling (3); hair loss (3); numbness/tingling (3); problem with concentration (3); problem with memory (3); aching muscles (3); aching joints (3); fatigue/lack of energy (3); hot flashes (3)
Akmansu (2025)^58^	Observational (Turkey)	Prostate	Radiotherapy	Symptomatic adverse event tracking	QLQ-CR29	EORTC	10	Anal pain (1); bloating (1); blood in stool (1); mucus in stool (1); gas incontinence (1); fecal incontinence (1); anal skin wound (1); daytime defecation (1); nighttime defecation (1); increase in bowel movements (1)
Anderson et al. (2023)^59^	Observational (United States)	Gynecological	Radiotherapy	Tracking symptomatic adverse events	N/A	PRO-CTCAE	Not specified	Gastrointestinal symptoms: diarrhea, flatulence, bowel incontinence, constipation; sexual health (pertinent); genitourinary symptoms (not specified); extremity swelling
Balachandran (2024)^61^	Observational (Denmark)	Gastrointestinal	Chemotherapy and surgery	Symptomatic adverse event tracking	QLQ-C30; FACIT-fatigue; Generalized Anxiety Disorder–7; Patient Health Questionnaire –9; Fear of Cancer Recurrence Inventory Short Form; Insomnia Severity Index	EORTC	6	Cognitive impairment (6)
Bjornholt (2025)^62^	Observational (Denmark)	Gynecological	Surgery	Symptomatic adverse event tracking	QLQ-C30; QLQ-EN24; Lymphedema QOL tool	EORTC	7	Tightness skin legs (1); tightness skin genitals (1); leg pain (1); swelling in genital area (1); swelling in groin (1); sore skin (1); pain in groin (1)
Brunner et al. (2023)^60^	Observational (Austria)	Breast	Chemotherapy	Efficacy, effectiveness or clinical benefit; tracking symptomatic adverse events	EORTC QLQ-C30; QLQ-BR23; body image scale	EORTC	4	Hair loss (4)
Chevallay (2025)^63^	Observational (UK)	Gastrointestinal	Surgery	Symptomatic adverse event tracking	N/A	EORTC	39	Number of items not specified: difficulties eating; problems with senses scale; anorexia; vomiting; nausea; weight loss; reflux scale; pain in chest; tiredness; diarrhea; constipation; hoarseness; jaundice; stool consistency; blood and mucus in stool; bowel movement frequency; anal pain; Global Health Status/QOL scale
Conti (2025)^64^	Observational (United States)	Blood cancers	Various cancer treatments	Other: financial toxicity	N/A	EORTC Other customization: 22 novel items	23	EORTC: financial difficulties (1) Novel: difficulty paying medical bills; delaying or foregoing medical care; financial worry; cost-coping strategies; treatment-related debt or bankruptcy
David (2024)^65^	Observational (Netherlands)	Various cancers	Not on active treatment	Symptomatic adverse event tracking	Fatigue Assessment Scale; HADS; Brief illness perception questionnaire	EORTC	1	Sleep problems (1)
den Hollander et al. (2022)^66^	Observational (Netherlands)	Gastrointestinal	Targeted therapy	Tracking symptomatic adverse events	EORTC QLQ-C30; EORTC SBQ	EORTC Other customizations: 1 self-developed item	1	Hand-foot syndrome (1)
Dierickx et al. (2022)^67^	Observational (Belgium)	Mixed disease, including cancer	Palliative care	Efficacy, effectiveness or clinical benefit (improvement in HRQOL domains)	EORTC QLQ-C30	EORTC Other customization: 1 item from other questionnaire; 2 self-developed items	4	Spiritual well-being (3); sexual health (1)
Gabbard (2024)^68^	Observational (United States)	Lung	Immunotherapy	Symptomatic adverse event tracking	QLQ-C30; PROMIS fatigue, anxiety and depression; Supportive Care Needs Survey Short Form–34	PRO-CTCAE Other customization: 1 item added from other questionnaire	21*	Constipation (1); fatigue (2); pain (3); cough (2); dyspnea (2); rash (1); dry skin (1); pruritus (1); muscle aches (3); joint pains (3); decreased appetite (2)
Haishan (2024)^69^	Observational (China)	Various cancers	Chemotherapy	Symptomatic adverse event tracking	N/A	PRO-CTCAE	38	Abdominal pain (3); anorexia (1); nausea (3); vomiting (2); constipation (3); diarrhea (2); cough (3); pain (3); headache (3); mucositis (3); anxiety (3); depression (2); insomnia (3); neuropathy (2); fatigue (2)
Han (2025)^70^	Observational (China)	Breast	Hormone therapy	Other: HRQOL	MDASI–Traditional Chinese Medicine	EORTC; FACIT Other customization: 14 items chosen from 2 item libraries and 1 other questionnaire	14	Not specified which item library: hot flashes (1); joint stiffness (1); arm swelling (1); hair loss (1); oral ulcers (1); diarrhea (1); urinary incontinence (1); abnormal vaginal discharge (1); vaginal dryness (1); vulvar itching (1); decrease in sexual interest or activity (1); sensitivity of the breast skin (1); breast tenderness (1); impaired concentration (1)
Heino et al. (2022)^71^	Observational (Finland)	Melanoma	Surgery	Efficacy, effectiveness or clinical benefit; tracking symptomatic adverse events	EORTC QLQ-C30; 15 dimension HRQOL instrument	EORTC	2	Edema–upper (1); edema–lower (1).
Horan (2024)^72^	Observational (United States)	Various cancers	Not on active treatment	Symptomatic adverse event tracking	EQ-5D-Y; PROMIS Meaning and Purpose scale	PRO-CTCAE	33	Stomach pain (3); constipation (3); mouth pain (3); nausea (3); fatigue (2); general pain (3); headache (3); numbness (2); worry (3); sadness (2); difficulty sleeping (3); cough (3)
Hsu et al. (2022)^73^	Observational (Taiwan)	Lung	Targeted therapy	Tracking symptomatic adverse events	Functional Assessment of Cancer Therapy-Epidermal Growth Factor Receptor Inhibitors–18;	PRO-CTCAE	7	Mouth/throat sores (1); cheilosis/cheilitis (1); skin dryness (1); acne (1); hair loss (1); itching (1); hand-foot syndrome (1)
Jackson-Carroll (2024)^74^	Observational (United States)	Melanoma	Immunotherapy	Symptomatic adverse event tracking	FACT-Melanoma; MDASI	MDASI	21	Lack of energy (1); itching (1); rash or skin changes (1); malaise/not feeling well (1); eye problems (1); skin problems (1); irritability (1); joint stiffness/soreness (1); headache (1); problems with feeling cold (1); fever or chills (1); muscle weakness (1); muscle soreness (1); problems with concentrating (1); weakness in arms or legs (1); problems with feeling hot (1); issues with balance (1); dizziness (1); mouth/throat sores (1); pain in the abdomen (1); problem with the teeth or gums (1)
Jacobs et al. (2022)^75^	Observational (United States)	Blood cancers	Chemotherapy	Tracking symptomatic adverse events	PROMIS Pediatric measures (physical function-mobility; pain interference; fatigue; depressive symptoms; psychological stress; anxiety)	Ped-PRO-CTCAE	38	Insomnia (3); pain (3); nausea (3); headache (3); anxiety (3); abdominal pain (3); not hungry (1); constipation (3); cough (3); mouth sores (3); diarrhea (2); vomiting (2); fatigue (2); sad (2); numbness (2)
Jakob et al. (2022)^76^	Observational (Germany)	Various cancers	Palliative care	Efficacy, effectiveness or clinical benefit (reduction in bone pain)	FACT-bone pain; VAS	FACIT	6	Pain management (2); time-stress impact on social, work, family life (4)
Joo Mi Park et al. (2023)^77^	Observational (South Korea)	Lung	Targeted therapy	Tracking symptomatic adverse events	Dermatology Life Quality Index	PRO-CTCAE Other customizations: self-developed item	11	Rash (1); hives (1); acne (1); nail ridging (1); nail discoloration (1); stretch marks (1); sensitivity to sunlight (1); skin dryness (1); itching (1); hair loss (1); skin darkening (1)
Kamimura (2024)^78^	Observational (Japan)	Various cancers	Chemotherapy	Symptomatic adverse event tracking	HADS	PRO-CTCAE	9	Anxious (3); discouraged (3); sad (3)
Lapen et al. (2022)^79^	Observational (United States)	Breast	Radiotherapy	Tracking symptomatic adverse events	Generalized Anxiety Disorder 2-item (screening tool); VAS for pain	Customization of PRO-CTCAE by splitting PRO-CTCAE item into 2	15	Pain (3); skin changes (6); breast enlargement or tenderness (3); swallowing (1); fatigue (2)
Lau (2025)^80^	Observational (United States)	Breast	Chemotherapy	Symptomatic adverse event tracking	National Health and Nutrition Examination Survey	PRO-CTCAE	9	Depression (3); discouraged (3); anxiety (3)
L’Hotta et al. (2023)^81^	Observational (United States)	Various cancers	Various cancer treatments	Other: change in social participation following cancer diagnosis Diagnostic (eg, scores were used to trigger referrals for support)	Community participation indicators; PROMIS-43	EORTC	1	Financial toxicity (1)
Lin (2024)^82^	Observational (China)	Gastrointestinal	Surgery	Symptomatic adverse event tracking	FACT-C	PRO-CTCAE	18	Decreased appetite (2); general pain (3); insomnia (2); fatigue (2); anxiety (3); discouragement (3); sadness (3)
McDowell (2025)^83^	Observational (Australia)	Head and neck	Chemoradiation	Symptomatic adverse event tracking	Chicago Priorities Scale modified	MDASI Other customization: 1 novel items	8	Sexual function preservation (1); chewing/swallowing food and drinks (1); pain (1); voice/speech problems (1); symptom interference with activities (1); fatigue (1); problems food taste (1); dry mouth (1)
Mittal (2025)^84^	Observational (Canada)	Lung	Chemotherapy/surgery	Symptomatic adverse event tracking	EQ-5D; Edmonton Symptom Assessment System	PRO-CTCAE	30	Shortness of breath (3); fatigue (3); decreased appetite (3); dizziness (3); cough (3); aching muscles (3); chest pain (3); abdominal pain (3); nausea (2); vomiting (2); constipation (1); diarrhea (1)
Muaddi (2025)^85^	Observational (United States)	Pancreatic	Surgery	Other: financial toxicity	FACIT financial distress	EORTC Other customization: 13 novel items	14	EORTC: financial difficulties (1) Novel: financial toxicity (13) (eg, impact of treatments; impact on employment; insurance coverage; cost coping strategies; financial planning; financial decision making)
Nash (2024)^86^	Observational (Australia)	Gynecological	Surgery	Efficacy, effectiveness or clinical benefit	N/A	Customization of EORTC: modified 7 item wordings and scales Other customization: 4 novel items	11	IN-PATSAT32 modified wording and response scale: treatment and medical follow-up (1); willingness of staff to listen (1); support given (1); information provision before the procedure (1); information provision on discharge (1); waiting time for results (1); speed of medical tests or treatment (1) Self-developed items: patient experience (4)
Nordhausen et al. (2022)^87^	Observational (Germany)	Various cancers	Radiotherapy	Tracking symptomatic adverse events and disease-related symptoms selected to construct electronic symptom monitoring tool (electronic PRO)	QLQ-C30; Questionnaire on Distress in Cancer Patients–R10	EORTC	19	Depression (1); skin problems (1); nausea (1); vomiting (1); pain (1); insomnia (1); appetite loss (1); constipation (1); diarrhea (1); tiredness (1); weakness (1); tumor specific (3-8)
Nugent (2024)^88^	Observational (UK)	Prostate	Radiotherapy	Symptomatic adverse event tracking	N/A	Customization of PRO-CTCAE: added symptoms not included in library Other customization: 1 novel item	19	Loose/watery stools (1); constipation (1); fecal incontinence (2); painful bowel movements* (2); pain (2); bleeding bowel movements* (2); blood in urine* (1); pain/burning when urinating (1); urinary urgency (2); urinary incontinence (2); erection problems (1); ejaculation problems (1) Novel: duration of hormone injections or tablets (1)
Nyrop (2025)^89^	Observational (United States)	Breast	Chemotherapy	Symptomatic adverse event tracking	FACIT-Fatigue; Mental Health Index	PRO-CTCAE	8	Fatigued (2); anxiety (3); depression (3)
O’Leary (2024)^90^	Observational (United States)	Various cancers	Surgery	Symptomatic adverse event tracking	N/A	PRO-CTCAE Other customization: 2 novel items	10	PRO-CTCAE: pain (3); shortness of breath (2); cough (2); other symptoms (1) Novel: redness (1); discharge at incision site (1)
Patel et al. (2023)^91^	Observational (United States)	Mixed diseases, including cancer	Stem cell transplantation	Efficacy, effectiveness or clinical benefit; tracking symptomatic adverse events Other: clinical monitoring development	FACT Bone Marrow Transplantation; PROMIS-10	Customization of PRO-CTCAE wording	41	Rash (1); itching skin (1); ulcers or sores on skin (1); pain or burning skin (1); problems with taste (1); decreased appetite (1); nausea (2); vomiting (1); heartburn (2); flatulence (1); bloating of abdomen (1); diarrhea (2); pain abdomen (3); lose control bowel movements (1); arm/leg swelling (2); aching muscles/joints (3); muscle weakness (2); pain (3); fatigue (2); insomnia (1); sad/unhappy (2); anxiety (2); memory/concentration (2); motivation (1); irritated (1); other symptoms (1); most bothersome symptom (1)
Patel (2024)^92^	Observational (United States)	Multiple myeloma	Various cancer treatments	Symptomatic adverse event tracking	N/A	EORTC; FACIT; PRO-CTCAE Customization of PRO-CTCAE: included more attributes (Presense or absense, orSeverity) than specified in PRO-CTCAE	30	PRO-CTCAE: anxiety (2); constipation (2); cough (2); decreased appetite (2); fatigue (2); general pain (2); insomnia (2); mouth/throat sores (2); muscle pain (2); nausea (2); numbness/tingling (2); sadness (2); shortness of breath (2); vomiting (2) FACT-GP5 (1); EORTC QLQ-C30 (1)
Roziner et al. (2023)^93^	Observational (multiple countries)	Breast	Various cancer treatments	Other: interrelation between somatic and emotional symptoms associated with breast cancer and its treatment	HADS; International Positive and Negative Affect Schedule Short Form	EORTC	33	QLQ-C30 Physical function scale (5) and role functioning scale (2); dyspnea (1); pain scale (2); fatigue scale (3); insomnia (1); appetite loss (1); nausea and vomiting scale (2); constipation (1); diarrhea (1) QLQ-BR23: systemic side effects (6) not complete scale; hair loss (1); arm symptom scale (3); breast symptom scale (4)
Ruan et al. (2023)^69^	Observational (China)	Various cancers	Chemotherapy	Tracking symptomatic adverse events	N/A	Ped-PRO-CTCAE	33	Abdominal pain (3); anorexia (2); nausea (2); vomiting (2); constipation (1); diarrhea (1); cough (2); pain (3); headache (3); mucositis (2); anxiety (3); depression (3); insomnia (2); neuropathy (2); fatigue (2)
Ruddy (2024)^94^	Observational (United States)	Head and neck	Chemotherapy	Symptomatic adverse event tracking	N/A	PRO-CTCAE	27	Dry mouth (1); difficulty swallowing (1); mouth or throat sores (2); skin cracking (1); voice changes (1); hoarse voice (1); tasting food (1); decreased appetite (2); nausea (2); vomiting (2); constipation (1); diarrhea (1); pain in the abdomen (3); cough (2); arm or leg swelling (3); fatigue (2); other symptoms (1)
Sakaguchi (2023)^95^	Observational (Japan)	Gastrointestinal	Chemotherapy	Symptomatic adverse event tracking	N/A	PRO-CTCAE	8	Mouth/throat sores (1); taste changes (1); rash (1); skin dryness (1); acne (1); hair loss (1); itching (1); nail ridging (1)
Schuler (2025)^96^	Observational (Australia)	Various cancers	Radiotherapy	Symptomatic adverse event tracking	N/A	PRO-CTCAE Other customization: 1 item added	Not specified	Not specified
Shawahna (2023)^97^	Observational (Palestine)	Multiple myeloma	Various cancer treatments	Efficacy, effectiveness or clinical benefit	QLQ-MY24	EORTC Other customization: 4 novel items	28	QLQ-MY24: disease symptoms (6); side effects of treatment (10); body image (1); future perspective (3); social support (4) Novel: patient satisfaction (4)
Smith (2024)^98^	Observational (United States)	Various cancers	Chemotherapy	Symptomatic adverse event tracking	FACT-G	PRO-CTCAE	10	Decreased appetite (1); nausea (1); vomiting (1); constipation (1); numbness or tingling in hands or feet (1); pain (1); mouth or throat sores (1); problems tasting food or drink (1); hand-foot syndrome (1)
Thorpe et al. (2022)^99^	Observational (United States)	Breast	Radiotherapy	Efficacy, effectiveness or clinical benefit; tracking symptomatic adverse events	PROMIS; QOL linear analogue self-assessment (specific questionnaires not reported)	PRO-CTCAE	14	Anxiety (1); insomnia (1); shortness of breath (2); cough (1); concentration (1); sad (1); skin burns (1); difficulty swallowing (1); decreased appetite (1); nausea (1); constipation (1); numbness or tingling in hands or feet (1); diarrhea (1)
Uneno (2024)^100^	Observational (Japan)	Various cancers	Chemotherapy	Efficacy, effectiveness or clinical benefit	N/A	EORTC; PRO-CTCAE	154	All EORTC QLQ-C30 items (30) All PRO-CTCAE (124)
van der Weijst et al. (2022)^101^	Observational (Belgium)	Lung	Various cancer treatments	Tracking symptomatic adverse events Other: disease-related symptoms	QLQ-C30	PRO-CTCAE	14	Symptom sets (items not specified) loss of appetite (1); pain (1); fatigue (1); dyspnea (1); cough (1); vomiting (1); nausea (1); dysphagia (1); diarrhea (1); constipation (1); anxiety (1); depression (1); memory problems (1); concentration problems (1)
Van de Wal (2024)^102^	Observational (Netherlands)	Gastrointestinal	Targeted therapy	Other: financial toxicity	QLQ-C30 Cancer Worry Scale; HADS	EORTC Other customization: 3 novel items	12	EORTC: physical condition or treatment causing financial difficulties (1); extra expenses that were difficult to pay (1); having extra expenses (1); lacking money to buy basic things (1); being in debt (1); changing one’s lifestyle because of financial difficulties (1); having less money to spend on oneself (1); experiencing problems paying regular expenses (1); having to borrow money or sell personal belongings (1) Novel: GIST specific concerns (3)
Walker et al. (2021)^103^	Observational (United States)	Various cancers	Palliative care	Tracking symptomatic adverse events and disease related symptoms Other: meaning of illness	Constructed Meaning Scale; MDASI brain tumor module; FACT-G; Centre for Epidemiological Studies Depression Scale Revised	MDASI	5	Radiating spine pain (1); numbness or tingling in the neck, trunk, arms, legs, or crotch (1); weakness in the arms and/or legs (1); loss of control of bowel and/or bladder (1); sexual function (1)
Wang et al. (2021)^104^	Observational (United States)	Blood cancers	Immunotherapy	Tracking symptomatic adverse events; other disease-related symptoms	EQ-5D; PROMIS-29; MDASI-Core and interference	MDASI	22	Diarrhea (1); weakness (1); speaking (1); lack of energy (1); feeling of malaise (1); bone aches (1); coughing (1); changes in sexual functioning (1); inability to eat (1); irritability (1); muscle soreness or cramping (1); difficulty paying attention (1); headache (1); poor balance or falling (1); fever or chills (1); dizziness (1); racing heartbeat or palpitations (1); swelling of the hands, legs, feet, abdomen, or around the eyes (1); tremors (1); difficulty swallowing (1); mouth/throat sores (1); rash (1)
Whisenant et al. (2022)^105^	Observational (United States)	Breast	Not on active treatment	Tracking symptomatic adverse events Other: functional interference	MDASI-Core and interference	MDASI	6	Diarrhea (1); constipation (1); mouth sores (1); skin rash (1); hair loss (1); coughing (1)
Withycombe et al. (2022)^106^	Observational (United States)	Various cancers	Chemotherapy	Tracking symptomatic adverse events Other: functional interference	PROMIS Pediatric instruments	Ped-PRO-CTCAE	38	Abdominal pain (3); anorexia (1); anxiety (3); constipation (3); cough (3); depression (2); diarrhea (2); fatigue (2); headache (3); insomnia (3); oral mucositis (3); nausea (3); pain (3); peripheral sensory neuropathy (2); vomiting (2)
Wujcik et al. (2022)^107^	Observational (United States)	Various cancers	Chemotherapy	Other: tracking symptomatic adverse events selected to construct electronic PRO	N/A	PRO-CTCAE	26	Fatigue (2); insomnia (2); pain (3); appetite loss (2); dyspnea (2); anxiety (3); nausea (2); depression (3); neuropathy (2); constipation (1); diarrhea (1); rash (1); mouth and throat sores (2)
Ya-Jung (2024)^108^	Observational (Taiwan)	Breast	Chemotherapy	Symptomatic adverse event tracking	N/A	MDASI	13	Pain (1); fatigue (1); nausea (1); disturbed sleep (1); distress/feeling upset (1); shortness of breath (1); difficulty remembering (1); lack of appetite (1); drowsiness (1); dry mouth (1); sadness (1); vomiting (1); numbness/tingling (1)

a PRO objective: PRO objective for the study categorized as measuring change (efficacy, effectiveness, or clinical benefit); tracking symptomatic adverse events (toxicities and tolerability); other (describe).

Abbreviations: EORTC = European Organisation for Research and Treatment of Cancer; EORTC QLQ-LC13 = EORTC Quality of Life Lung-specific module; EORTC QLQ-BR23 = EORTC Quality of Life Breast-specific module; EORTC QLQ-C30 = EORTC Quality of Life Core questionnaire; EORTC QLQ-CIPN20 = EORTC Quality of Life CIPN module; EORTC QLQ-MY20 = EORTC Quality of Life Multiple Myeloma module; EORTC-SBQ = EORTC Symptom-based questionnaire; EQ-5D = EuroQol-5 Dimension; EQ-5D-5L = EuroQol-5 Dimension 5-level; FACIT = Functional Assessment of Chronic Illness Therapy; FACT = Functional Assessment of Cancer Therapy; FACT-G = FACT-General; GIST = gastrointestinal stromal tumor; HRQOL = health-related quality of life; MDASI = MD Anderson Symptom Inventory; MDASI LC = MDASI Lung Cancer; Ped-PRO-CTCAE = Pediatric PRO-CTCAE; PRO = patient-reported outcome; PRO-CTCAE = Patient-Reported Outcomes version of the Common Terminology Criteria for Adverse Events; PROMIS = Patient-Reported Outcomes Measurement System; QLQ-CR29 = Colorectal; QLQ-EN24 = endometrial; QLQ-MY24 = Multiple Myeloma; VAS = visual analogue scale; HADS = Hospital Anxiety and Depression Scale; IN-PATSAT32 = EORTC in-patient satisfaction with cancer care questionnaire; N/A = Not Applicable.

**Table 2.** Study characteristics and item library use classified by study design.

Category	Trials/feasibility studies (n = 25)	Observational studies (n = 53)	All studies (N = 78)
PRO item library used			
PRO-CTCAE (including Ped-PRO-CTCAE)	18	26	44
EORTC	2	17	19
MDASI	1	6	7
PRO-CTCAE and EORTC	1	1	2
EORTC and FACIT and PRO-CTCAE	1	1	2
FACIT and PRO-CTCAE	1	0	1
EORTC and FACIT	0	1	1
FACIT	0	1	1
PROMIS	1	0	1
Country			
United States	18	23	41
China	1	4	5
Japan	2	3	5
Australia	0	3	3
Denmark	1	2	3
Netherlands	0	3	3
Belgium	0	2	2
Canada	1	1	2
Germany	0	2	2
Taiwan	0	2	2
United Kingdom	0	2	2
Austria	0	1	1
Finland	0	1	1
Multinational	0	1	1
South Korea	0	1	1
Turkey	0	1	1
Thailand	1	0	1
Iceland	1	0	1
Palestine	0	1	1
Funding			
Academic	15	46	61
Commercial	6	4	10
Mixed	4	3	7
Cancer type			
Mixed cancer population	6	15	21
Breast	5	10	15
Gastrointestinal	5	6	11
Lung	3	5	8
Gynecological	3	3	6
Blood cancers	1	3	4
Prostate	1	2	3
Multiple myeloma	1	2	3
Mixed disease including cancer	0	2	2
Melanoma	0	2	2
Head and neck	0	2	2
Pancreatic	0	1	1
Age			
18 years and older	25	48	73
Younger than 18 years	0	5	5
Treatment type			
Chemotherapy	9	15	24
Surgery	2	7	9
Radiotherapy	1	7	8
Various cancer treatments	2	6	8
Chemoradiation	4	1	5
Targeted therapy	1	4	5
Immunotherapy	2	3	5
Palliative care	1	3	4
Not on active treatment	0	3	3
Hormone	1	1	2
Chemotherapy and surgery	0	2	2
Chemotherapy andtargeted therapy	1	0	1
Stem cell transplantation	0	1	1
Immunotherapy and targeted therapy	1	0	1
PRO objective of the study			
Symptomatic adverse event tracking^a^	21	42	63
Efficacy, effectiveness, clinical benefit^a^	10	9	19
Other	3	6	9
Used with PRO measures			
Yes	16	36	52
Type item library customization			
Single/multiple single items	20	45	65
Scale(s) and multiple item(s)	2	6	8
Not specified	2	1	3
Scale(s) and single item(s)	1	1	2
Unexpected issues item(s)			
Yes	6	4	10
Electronic completion available			
Yes	12	28	40
Method to reduce respondent burden reported			
Yes	3	3	6
Order issues reported in methods			
Yes	1	1	2

Abbreviations: EORTC = European Organisation for Research and Treatment of Cancer; FACIT = Functional Assessment of Chronic Illness Therapy; MDASI = MD Anderson Symptom Inventory; Ped-PRO-CTCAE = Pediatric PRO-CTCAE; PRO-CTCAE = Patient-Reported Outcomes version of the Common Terminology Criteria for Adverse Events; PROMIS = Patient-Reported Outcomes Measurement Information System.

a Not mutually exclusive.

Most studies were conducted in the United States, with the PRO-CTCAE representing the most frequently used PRO item library. In 63 of the 78 studies, symptomatic adverse event tracking was part of the PRO objectives. The majority of studies were academically funded (61 of 78)^33-37^^,^^39^^,^^40^^,^^43-47^^,^^49-51^^,^^55^^,^^56^ and conducted with adult populations (73 of 78).^32-68^^,^^70^^,^^71^^,^^73^^,^^74^^,^^76-105^^,^^107^^,^^108^ Studies that included participants from mixed cancer types were the most common (21of 78). The largest single-disease group studied was breast cancer (15 of 78). Chemotherapy-based treatments (32 of 78) followed by surgery (9 of 78) were the most common treatment types studied, although a range of other therapies were investigated. Electronic completion of PRO measures was reported as available in 40 of 78 studies.^32^^,^^33^^,^^39^^,^^40^^,^^43-48^^,^^51^^,^^53^^,^^61^^,^^62^^,^^64-66^^,^^69^^,^^75^^,^^76^^,^^79^^,^^81-83^^,^^85-93^^,^^96^^,^^99^^,^^100^^,^^102^^,^^104^^,^^106^^,^^107^

The majority of studies reported the item library they used within the publication abstract (66 of 78), whereas 22 specified the items selected from the item library in the abstract. Thirteen abstracts did not specify the items but referred to customization using terms such as supplemented, prespecified, selected, or modified. Of the 78 articles, 43 did not refer to customization in the abstract; this was evident only during full text review.

### Item libraries that were used 

Five item libraries were identified: PRO-CTCAE (49 of 78), EORTC (24 of 78), MDASI (7 of 78) FACIT (5 of 78), and PROMIS (1 of 78) (Table 2). The PRO-CTCAE (including the pediatric version [Ped-PRO-CTCAE]) was the most frequently used item library across all studies: trials and feasibility studies (18 of 25) and observational studies (26 of 53). This was followed by the EORTC item library, which was used in 24 studies: trials and feasibility studies (4 of 15) and observational studies (20 of 78). Six studies used 2 or more PRO item libraries.

### The contexts where item lists were used

We categorized the studies according to contexts of use, as described in Table 3, and found that uses were not mutually exclusive. Some studies were assigned to more than 1 category, especially among the trials and feasibility studies. Of the trials and feasibility studies, 17 of 25 were early phase (phase 1 or 2).^33-36^^,^^38-41^^,^^43^^,^^45-52^ Of the 78 studies, 15 looked at rare diseases including pancreatic cancer, platinum resistant or refractory epithelial ovarian cancer, relapsed or refractory multiple myeloma, anal cancer, gastrointestinal stromal tumor, acute graft-vs-host disease, and leptomeningeal metastases.^35^^,^^40^^,^^42^^,^^45^^,^^48-50^^,^^66^^,^^83^^,^^85^^,^^91^^,^^92^^,^^94^^,^^97^^,^^103^ Of the 78 studies, 19 examined the effects of a novel treatment, including targeted therapy, intensity-modulated proton therapy, or oral cannabidiol.^32^^,^^38^^,^^41^^,^^42^^,^^48^^,^^50^^,^^51^^,^^53^^,^^54^^,^^56^^,^^59^^,^^66^^,^^68^^,^^73^^,^^74^^,^^77^^,^^88^^,^^96^^,^^104^ In addition, 20 of 78 studies used item lists to support clinical monitoring or as part of a supportive care intervention, with 6 of these using an item library to develop a symptom list for electronic PRO monitoring in a clinical care context.^35-37^^,^^39^^,^^40^^,^^43-47^^,^^58^^,^^60^^,^^63^^,^^87^^,^^90^^,^^91^^,^^94^^,^^100^^,^^103^^,^^107^ Fourteen studies were not described by the “context of use” categories above.^55^^,^^57^^,^^67^^,^^69^^,^^71^^,^^75^^,^^76^^,^^79^^,^^81^^,^^93^^,^^99^^,^^101^^,^^105^^,^^106^

**Table 3.** Context of use.

Category	Trials and feasibility studies (n = 25)	Observational studies (n = 53)	Totals (N = 78)
Early-phase trial or feasibility study context of use	17	N/A	17
Rare diseases context of use	7	8	15
Novel treatment context of use	10	9	19
Clinical monitoring or supportive care intervention context of use	10	10	20
Electronic patient-reported outcome context of use	6	3	4
Studies not described by context of use categories above	1	13	14
Selection methods were reported^a^	16	22	38
Reported more than 1 selection method^a^	10	8	18
Selection method: literature, evidence and core symptom sets^a^	14	18	32
Selection method: health-care professionals consulted^a^	3	8	11
Selection method: patients or patient representatives consulted^a^	3	5	8

a Not mutually exclusive.

### Item list content 

The types of issues and the number of items included in each study are listed in Table 1. The issues captured ranged between disease- and treatment-related symptoms and the impact of cancer on a function or aspect of everyday life. Items lists derived from the PRO-CTCAE tracked patient-reported symptomatic adverse events. The lists used varied in length from 1 to 45 items, with a median of 14 items and an interquartile range of 18 items. As described in Table 2, the majority (52 of 78) of item lists were used in studies alongside standard PRO measures.^33-35^^,^^37^^,^^39-43^^,^^46^^,^^47^^,^^49^^,^^51^^,^^53^^,^^54^^,^^56^^,^^58^^,^^60-62^^,^^65-68^^,^^70-85^^,^^87^^,^^89^^,^^91^^,^^93^^,^^98^^,^^99^^,^^101-106^ Most (65 of 78) item lists were comprised of multiple single items^32-34^^,^^36-38^^,^^40^^,^^41^^,^^43^^,^^45^^,^^46^^,^^48-57^^,^^60-62^^,^^64-73^^,^^75-92^^,^^94-96^^,^^98^^,^^99^^,^^101-103^ (Table 2), with 10 studies including multi-item scales from existing validated questionnaires as part of their PRO measurement strategy.^39^^,^^42^^,^^44^^,^^58^^,^^63^^,^^74^^,^^93^^,^^97^^,^^100^^,^^108^ Three studies did not specify the composition of the item list.^35^^,^^47^^,^^59^

Some studies provided clear rationales as to why additional items were necessary. For example, Jakob et al.^76^ investigated PROs in patients with breast or prostate cancer receiving antiresorptive therapy for bone metastases and supplemented the Functional Assessment of Cancer Therapy–Bone Pain with 6 items relevant to their research questions but not included in the Functional Assessment of Cancer Therapy–Bone Pain. These items captured pain management and time-stress impact on work, social, and family life. Brunner et al.^60^ selected 4 items from the EORTC item library to collect patient perspectives of hair loss, as these items were not included in any of the other measures used. Patel et al.^91^ selected items to capture patient perspectives of graft-vs-host disease symptoms because no validated PRO measure was available for this condition. Walker et al.^103^ developed a modified version of the MDASI-BT (brain tumor module) to investigate symptom burden in patients with leptomeningeal metastasis who may have disease involving both the brain and spine. The modification involved adding 5 items from the MDASI-SP (spine tumor module) to the MDASI-BT.

Ten studies (6 trials or feasibility studies^32^^,^^33^^,^^44^^,^^48-50^ and 4 observational studies^86^^,^^90^^,^^91^^,^^94^) reported including item(s) to capture unexpected issues (Table 2). Typically, these aim to provide a space where patients can add free text (or use predictive text responses in an electronic PRO system) to report symptoms or problems they have experienced that are not included in the PRO measures or item lists provided.

### How item libraries were used

Most (72 of 78) studies used a single item list, which was derived from 1 item library. Four studies used 2 libraries to create 2 item lists. Knoerl,^44^ Hummel,^41^ and Uneno^100^ specified the source and content of the 2 item lists, however, Han^70^ appears to have created a single item list from 2 item libraries. Two studies reported using items from 3 item libraries, and both specified their sources.^42^^,^^92^

Most studies used the original item wording from the respective item library. However, some studies showed evidence of further customization when creating an item list. These customizations took 2 forms: making changes to items derived from the item library (7 of 78); or adding items from other sources, such as other named PRO measures or self-developed items (20 of 78). The first type of customization was reported within studies that made changes to item wording or added new items while retaining the original item library format. For example, Lapen et al.^79^ compared patient- and clinician-reported acute radiation toxicities and included new, detailed questions about the effects of radiation on the skin, while retaining the PRO-CTCAE format. They also modified wording of existing PRO-CTCAE items, for example, splitting the item on “breast enlargement or tenderness” to create 2 individual items, as well as modifying the item wording for other items, including “skin coloration” instead of “skin darkening.” Another example comes from Adesoye et al.,^57^ who added new items by using different combinations of the attributes provided by the PRO-CTCAE system. For example, an item on “vomiting-interference” was included despite not being available in the PRO-CTCAE item library.

The second type of customization was reported in studies that added items from sources other than one of the PRO item libraries. For example, Basch et al.^33^ included selected symptoms from the PRO-CTCAE and added 4 questions about bladder and sexual function from existing validated PRO measures (eg, 1 question about bladder function was added from the Prostate Health-Related QOL questionnaire^109^ and another from the International Prostate Symptom Score).^110^ The 4 additional questions were presented separately from the PRO-CTCAE at different time points of the trial.

In addition to the customizations described above, it should be noted that there was an underreporting of item library usage by some studies, whereby reference to the item library was omitted or unclear (21 of 78). For example, Martin et al.^49^ selected the “Future Perspective” scale, consisting of 4 items from the EORTC Quality of Life Multiple Myeloma Questionnaire–20, without referencing the use of the EORTC item library. Additionally, Roziner et al.^93^ customized 2 EORTC questionnaires to create their item list and did not directly reference using the EORTC item library.

### Methods for item selection and minimizing respondent burden

There was variation across the studies in the methods used to select the items for inclusion in the item list, with 38 studies reporting their methods (Table 3).^32-34^^,^^37^^,^^40-49^^,^^53^^,^^54^^,^^58^^,^^63^^,^^64^^,^^66^^,^^67^^,^^69^^,^^70^^,^^74^^,^^75^^,^^78^^,^^79^^,^^85^^,^^87^^,^^91^^,^^93^^, ^^95^^,^^96^^,^^102^^,^^104^^,^^106^^,^^107^ The methods used were not mutually exclusive, and some studies reported using more than 1 method.^33^^,^^37^^,^^40^^,^^43^^,^^45^^, ^^46^^,^^48^^,^^49^^,^^53^^,^^54^^,^^63^^,^^64^^,^^66^^,^^67^^,^^70^^,^^87^^,^^91^^,^^107^ Selection from literature sources was cited in 14 of 25 trials or feasibility studies^33^^,^^34^^,^^40-49^^,^^53^^,^^54^ and 18 of 53 observational studies^58^^,^^63^^,^^64^^,^^66^^,^^67^^,^^69^^,^^70^^,^^75^^,^^78^^,^^79^^,^^85^^,^^87^^,^^95^^,^^96^^,^^102^^,^^106^^,^^107^ and included the use of investigational brochures, previous empirical work by the study team, reviews, and mapping to published core symptoms sets.

The views of health-care professionals were sought in 3 trials^32^^,^^37^^,^^46^ and 8 of 53 observational studies.^66^^,^^67^^,^^70^^,^^74^^,^^87^^,^^91^^,^^93^^,^^107^ Patient or patient representative feedback was collected in 3 trials^33^^,^^37^^,^^53^ and 5 observational studies^63^^,^^66^^,^^67^^,^^91^^,^^104^ (8 of 78). Of the 8 studies, 3 reported the number of patients involved in item selection. In a study by Patel et al.,^91^ the selection of survey measures and items was informed by discussions with health and social care professionals, including doctors, nurses, and social workers. Subsequently, the survey was pilot-tested with the first 10 patients to complete the survey to identify redundant, confusing, or irrelevant questions and effects of item order. However, they did not report how the survey was modified in response to the pilot-test findings. Dierickx et al.^67^ developed 2 questionnaires: one for clients from palliative day care centers and the other for family caregivers. They developed the survey through consultation with a multidisciplinary team and pilot tested the questionnaires with 3 patients and 2 caregivers. Wang et al.^104^ selected items from the MDASI symptom library based on clinician expertise and the published literature related to chimeric antigen receptor T-cell therapy and interviewed 21 patients undergoing this therapy to confirm content validity. Three trials^33^^,^^37^^,^^59^ involved patient representatives or advocates in the conduct of the trial, which included consultation in relation to item selection. However, the number of representatives who participated in item selection was not reported.

Respondent burden is defined as the degree to which a survey respondent perceives participation in a study as difficult, time-consuming, or stressful.^111^ Of the 78 studies, 6 reported the methods they took to reduce respondent burden,^37^^,^^53^^,^^56^^,^^67^^,^^87^^,^^91^ which included avoiding duplication of concepts, adjustment of the PRO assessment schedule, and piloting the item list. In one study, symptomatic adverse events were not duplicated in the item list if they were already captured within another questionnaire.^37^ In another study, Yeung et al.^56^ reduced the number of PRO measures administered at week 3 of radiotherapy treatment when treatment burden was likely to be at its highest.

## Discussion

To our knowledge, this is the first systematic review to investigate both the use of PRO item libraries and the implementation of PRO item lists within cancer-related research studies. The increasing use of PRO item libraries over the course of the reviewed period highlights the timely nature of this review. We identified item libraries used across different research contexts, including feasibility studies, early-phase and late-phase clinical trials, and observational studies. Five cancer PRO item library measurement systems were used to create PRO item lists. The most common application of the item lists was to assess symptomatic adverse events (within trials or feasibility studies and observational research studies). We found that the PRO-CTCAE (which specializes in this type of usage) was the most frequently used PRO item library.^11^^,^^25^ Item lists were usually included as a component of PRO measurement strategies alongside validated PRO measures.

The literature underscores the value of implementing PRO item lists within clinical trials, particularly those associated with the evaluation of medical products, ^11^^,^^112^ highlighting their relevance within novel treatment, rare cancer, early-phase trial, and symptom monitoring contexts.^10^ The use of item lists in these contexts was supported, as we found that trial and feasibility studies could be classified into 1 or more of these categories. This suggests that item lists are a useful addition in early-phase trials when less is known about the possible impact of a treatment, in rare cancers where standard PRO measures may not be available, and in the study of novel treatments where concepts (eg, symptoms) may be missing from standard PRO measures. The use of item lists in observational research was found to be well established. Here, they were used to investigate patient perspectives across a range of common cancers or established treatment types and where applicable (to support the study’s aims) supplemented validated PRO measures. Currently there is limited guidance available regarding how item lists or item libraries are best used within oncology research and real-world settings.^10^^,^^113^

Extant literature cautions against modifications to the wording of existing items;^10^ however, we found evidence of further customization of item library items such as adding novel items or editing wording but retaining the format found in the item library. Studies also included other types of customizations, such as using self-developed items or items from existing validated questionnaires outside of the PRO item libraries used. These additional customizations indicate that item libraries may not be fully comprehensive and may not cover all relevant concepts required to meet all study aims and objectives. This suggests the need for a process whereby item libraries can be regularly assessed to determine their coverage of concepts. Increasing access to item libraries and familiarizing users with their content should be encouraged to mitigate the need for additional customization when it can be avoided.

In our review, we found that some studies did not specify their use of an item library. As suggested by Piccinin et al.,^10^ greater transparency in describing item library and item list usage may help minimize measurement or reporting bias along with threats to the reliability or integrity of the study. Future studies should consider including information about content and methods used to develop PRO item lists within trial or study protocols and in the reporting of study outcomes. It is important to note that data included within this review may predate the publication of any such guidance and partially explain the variation in reporting across the studies.

Consistent with existing recommendations,^10^ we found a range of methods used to select items. More than half of the studies reported using at least 1 specific method to select the included items. We found trials and feasibility studies generally provided more information about item selection than observational studies.^33^ Methods fell into 3 categories: (1) literature reviews, for example investigator brochures, previous empirical work, or use of core symptoms set lists to guide item selection; (2) health-care professional consultation; and (3) patient or patient representative views, for example, patient representative involvement, interviews, or a pilot study. Use of more than 1 method to develop an item list and enabling patients to report additional symptoms are valuable ways to increase content validity and minimize possible bias.^9^^,^^25^ Moreover, reporting the methods used to select items increases transparency by providing evidence that cherry-picking has been avoided.^10^

The literature suggests that methods to detect unexpected issues (eg, using free-text responses) are especially useful in early-phase trials where less may be known about the issues faced.^26^ We found these approaches were used by 3 of the 15 early-phase trials and feasibility studies, which used this information to support PRO item selection in later phases of the trial.^33^^,^^48^^,^^49^ This is potentially a growth area for new item development as the methods of analysis of free-text responses continue to develop.^114^

In terms of limitations, because of the complex and novel nature of the review topic, studies that used an item library but did not refer to any type of customization in the abstract may have been missed. In addition, we did not search for all available PRO item libraries by name. However, measures were taken to mitigate publication bias, primarily through the development of a broad search strategy to ensure that studies that used an item library approach were included, even when the abstract did not specify how the items were sourced. Further, we took a “benefit of the doubt” approach at the title and abstract screening stage and completed full-text screening on a large number of articles. Despite these efforts, it is possible that some articles may have been missed. In some publications, for example, word count constraints might account for underreporting of some aspects of methods associated with item list use.

A consequence of the benefit of the doubt approach was that it was necessary to review a high number of full texts, prompting the decision to limit the review period to publications over a 4-year period. However, the date range in which some studies collected data spanned several years, with some going as far back as 2004. It was not possible to extract some of the planned data^31^ such as information about the validity, reliability, or responsiveness to a change of the item lists as these data were not reported in the included articles. A future systematic review of methodological or development publications may reveal more about approaches used to evaluate psychometric properties.

To our knowledge, this review is novel as we examined how item libraries are used across oncology research. It is the first systematic review examining the use of item lists across a range of item libraries. Furthermore, it has broad relevance, including practices in observational studies, early-phase trials, and feasibility studies, rather than limiting the focus to randomized controlled cancer trials only. We developed a robust data extraction framework a priori, which facilitated the analysis of a heterogenous sample of study designs and allowed us to identify several areas that could be addressed in future guidance.

There are areas where further review would be useful, including an evaluation of the use of item lists in real-world settings and an exploration of possible trends toward symptom monitoring as indicated in early-phase trials in this systematic review. Estimates of the tolerability of treatments in early-phase and late-phase trials using PROs alongside clinician toxicity reporting is likely to be an area of growth as regulatory recommendations take effect.^11^^,^^26^ A review of clinical care developments would require a broader review to include gray literature such as clinical trials databases, audits, and service improvements, as well as articles. A systematic review of current study protocols would also shed light on when and how item libraries are used, potentially highlighting the impact of available recommendations and recent regulatory guidance. Guidelines for reporting PRO item lists should be developed for clinical trials and routine care, building on existing evidence.^10^ These guidelines could follow the structure of established PRO reporting standards such as CONSORT-PRO (Consolidated Standards of Reporting Trials - Patient Reported Outcomes) and SPIRIT-PRO (Standard Protocol Items: Recommendations for Interventional Trials - Patient Reported Outcomes), which have been successfully implemented in practice. Adopting a similar framework would support consistent monitoring, improve transparency, and enhance the quality of reporting. In particular, the systematic inclusion of item lists as an appendix or as part of supplementary material for protocols or publications would provide valuable information on the included concepts. PRO item library developers may wish to consider how registered individuals may be able to easily access such a list for publication if not already available.

In conclusion, these findings highlight a need for additional flexibility in PRO measurement within different research settings, supported by PRO item libraries. However, as evidenced in the current literature, it is important that methodological guidelines and a rigorous framework for reporting are developed to increase transparency, reduce inconsistencies, and promote best practices when it comes to item library usage. Findings identified in this review will be addressed in a Delphi survey aimed at developing international guidelines to support PRO item library implementation in clinical trials and practice.

## Supplementary Material

djag039_Supplementary_Data

## Data Availability

The data underlying this article are available in the article and in its online supplementary material.
