# Supplementary material for: Systematic review of patient-reported outcome item libraries in cancer research: an EORTC Quality of Life Group study
Source: J Natl Cancer Inst. 2026 Feb 16;118(6):971–96. doi: 10.1093/jnci/djag039 (PMC13247343; doi:10.1093/jnci/djag039)
Supplement: djag039_Supplementary_Data [file djag039_supplementary_data.docx]

### Supplementary methods: Data categories and definitions

| DATA EXTRACTED | CATEGORY DEFINITION |
| --- | --- |
| Author, publication date | As reported in paper: first author and publication date |
| Title | As reported in paper |
| Country | As reported in paper |
| Funding | As reported in paper: categorised as academic, commercial, or mixed |
| Data collection date | As reported in paper |
| Study design | Trial or feasibility study or observational study |
| Cancer type | Broad cancer types categorised by body system, or various cancers, or mixed diseases (including cancer) |
| Intervention or treatment | Broad category by intervention or treatment type |
| Age of participants | As reported in paper: categorised adult or paediatric |
| Trial phase | Trials only |
| PRO primary or secondary endpoint | Trials only |
| PRO objectives | Classified as efficacy (including effectiveness or clinical benefit) or tracked symptomatic adverse events (including toxicities or tolerability) or other. |
| PRO measurement system | PRO-CTCAE (inc. Ped-PRO-CTCAE); EORTC; MDASI; FACIT; PROMIS |
| PRO questionnaires | Name of complete, validated PRO questionnaires reported |
| PRO administration mode | Electronic, paper or both reported |
| PRO item library | PRO-CTCAE (inc. Ped-PRO-CTCAE); EORTC; MDASI; FACIT; PROMIS |
| Formal use of PRO items | Defined as cases where the wording, recall period, or response scale of the item was unchanged. |
| Name and number of items | Name and frequency count. Median and inter-quartile range calculated |
| Other customisations | Inclusion of self-developed items or items of unknown source |
| Free-text option | Write In three Symptoms/Problems' (WISP) or other methods for patient select symptoms in own words |
| Item selection | Did methods report item selection. Broadly categorised into literature, involved healthcare professionals, involved patients or patient representatives |
| Item order | Did methods report whether item order was addressed in the methods |
| Respondent burden | Did the study report whether respondent burden was addressed in the methods |
| Mode of collection | Electronic, paper, both or not specified |
| Context of use | Categories not mutually exclusive: use in early phase trials; for novel treatments; for rare cancers; clinical monitoring; other |
| FDA Guidance (2021) | Was FDA (2021) guidance referenced |
| Abstract | Item library use or customisation reported in abstract |

### Supplementary methods: Database search terms

| **Medline search conducted 02/10/2023** | **Embase search conducted 02/10/2023** | **CINAHL search conducted 02/10/2023** |
| --- | --- | --- |
| 1 exp Neoplasms/  2 cancer*.tw.  3 oncology.tw.  4 neoplasm*.tw.  5 carcinoma*.tw.  6 leuk?emia*.tw.  7 lymphoma*.tw.  8 malignan*.tw.  9 sarcoma*.tw.  10 glioblastoma*.tw.  11 Mesothelioma*.tw.  12 tumo?r*.tw.  13 metasta*.tw. 626065  14 adenocarcinom*.tw.  15 adeno-carcinom*.tw.  16 or/1-15 [cancer]  17 (Librar* adj3 (Item or Items or PRO or PROM or Symptom or EORTC or PRO-CTCAE or PROMIS or FACIT or MDASI or Questionnaire* or Question or Questions or Sub-scale*)).mp.  18 (Modified adj3 (Item or Items or PRO or PROM or Symptom or EORTC or PRO-CTCAE or PROMIS or FACIT or MDASI or Questionnaire* or Question or Questions or Sub-scale*)).mp.  19 (Additional adj3 (Item or Items or PRO or PROM or Symptom or EORTC or PRO-CTCAE or PROMIS or FACIT or MDASI or Questionnaire* or Question or Questions or Sub-scale*)).mp.  20 (Supplement* adj3 (Item or Items or PRO or PROM or Symptom or EORTC or PRO-CTCAE or PROMIS or FACIT or MDASI or Questionnaire* or Question or Questions or Sub-scale*)).mp.  21 ((Bespoke adj3 (Item or Items or PRO or PROM or Symptom or EORTC or PRO-CTCAE or PROMIS or FACIT or MDASI or Questionnaire* or Question or Questions)) or Sub-scale*).mp.  22 (Customi?ed adj3 (Item or Items or PRO or PROM or Symptom or EORTC or PRO-CTCAE or PROMIS or FACIT or MDASI or Questionnaire* or Question or Questions or Sub-scale*)).mp.  23 (Item* adj3 (PRO or PROM or Symptom or EORTC or PRO-CTCAE or PROMIS or FACIT or MDASI or librar* or bank* or set* or selection or sub-scale*)).mp.  24 Patient reported outcome measurement information system.mp.  25 Patient reported outcome* librar*.mp.  26 Searchable librar*.mp.  27 Patient Reported Outcome item*.mp.  28 (European Organisation for Research and Treatment of Cancer item library).mp.  29 MD Anderson Symptom Inventory.mp.  30 Symptom librar*.mp.  31 Functional Assessment of Chronic Illness Therapy searchable library.mp.  32 Patient Reported Outcomes version of the Common Terminology Criteria for Adverse Events.mp.  33 Patient reported outcome* item set.mp.  34 17 or 18 or 19 or 20 or 21 or 22 or 23 or 24 or 25 or 26 or 27 or 28 or 29 or 30 or 31 or 32 or 33  35 16 and 34  36 limit 35 to English language  37 limit 36 to yr="2020 -Current" | 1 exp Neoplasms/  2 cancer*.tw.  3 oncology.tw.  4 neoplasm*.tw.  5 carcinoma*.tw.  6 leuk?emia*.tw.  7 lymphoma*.tw.  8 malignan*.tw.  9 sarcoma*.tw.  10 glioblastoma*.tw.  11 Mesothelioma*.tw.  12 tumo?r*.tw.  13 metasta*.tw.  14 adenocarcinom*.tw.  15 adeno-carcinom*.tw.  16 or/1-15 [cancer]  17 (Librar* adj3 (Item or Items or PRO or PROM or Symptom or EORTC or PRO-CTCAE or PROMIS or FACIT or MDASI or Questionnaire* or Question or Questions or Sub-scale*)).mp.  18 (Modified adj3 (Item or Items or PRO or PROM or Symptom or EORTC or PRO-CTCAE or PROMIS or FACIT or MDASI or Questionnaire* or Question or Questions or Sub-scale*)).mp.  19 (Additional adj3 (Item or Items or PRO or PROM or Symptom or EORTC or PRO-CTCAE or PROMIS or FACIT or MDASI or Questionnaire* or Question or Questions or Sub-scale*)).mp.  20 (Supplement* adj3 (Item or Items or PRO or PROM or Symptom or EORTC or PRO-CTCAE or PROMIS or FACIT or MDASI or Questionnaire* or Question or Questions or Sub-scale*)).mp.  21 ((Bespoke adj3 (Item or Items or PRO or PROM or Symptom or EORTC or PRO-CTCAE or PROMIS or FACIT or MDASI or Questionnaire* or Question or Questions)) or Sub-scale*).mp.  22 (Customi?ed adj3 (Item or Items or PRO or PROM or Symptom or EORTC or PRO-CTCAE or PROMIS or FACIT or MDASI or Questionnaire* or Question or Questions or Sub-scale*)).mp.  23 (Item* adj3 (PRO or PROM or Symptom or EORTC or PRO-CTCAE or PROMIS or FACIT or MDASI or librar* or bank* or set* or selection or sub-scale*)).mp.  24 Patient reported outcome measurement information system.mp.  25 Patient reported outcome* librar*.mp.  26 Searchable librar*.mp.  27 Patient Reported Outcome item*.mp.  28 (European Organisation for Research and Treatment of Cancer item library).mp.  29 MD Anderson Symptom Inventory.mp.  30 Symptom librar*.mp.  31 Functional Assessment of Chronic Illness Therapy searchable library.mp.  32 Patient Reported Outcomes version of the Common Terminology Criteria for Adverse Events.mp.  33 Patient reported outcome* item set.mp.  34 17 or 18 or 19 or 20 or 21 or 22 or 23 or 24 or 25 or 26 or 27 or 28 or 29 or 30 or 31 or 32 or 33 37266  35 16 and 34  36 limit 35 to English language  37 limit 36 to yr="2020 -Current"  38 limit 37 to "remove medline records" | 1 (MH “Neoplasms”)  2 TX neoplasms  3 (MH “Cancer Patients”  4 (MH “Cancer Survivors”  5 TX cancer patients  6 TX cancer survivors  7 TX cancer*  8 TX oncology  9 TX tum?r*  10 TX sarcoma*  11 TX carcinoma*  12 TX leu?emia*  13 TX lymphoma*  14 TX mesothelioma*  15 TX glioblastoma*  16 TX metasta*  17 TX adenocarcinoma*  18 S1 OR S2 OR S3 OR S4 OR S5 OR S6 OR S7 OR S8 OR S9 OR S10 OR S11 OR S12 OR S13 OR S14 OR S15 OR S16 OR S17  19 (librar*n3 (item or items or PRO or PROM or symptom or EORTC or PRO-CTCAE or PROMIS or FACIT or MDASI or questionnaire* or question or questions or sub-scale*))  20 (modified n3 (item or items or PRO or PROM or symptom or EORTC or PRO-CTCAE or PROMIS or FACIT or MDASI or questionnaire* or question or questions or sub-scale*))  21 (additional n3 (item or items or PRO or PROM or symptom or EORTC or PRO-CTCAE or PROMIS or FACIT or MDASI or questionnaire* or question or questions or sub-scale*))  22 (supplement* n3 (item or items or PRO or PROM or symptom or EORTC or PRO-CTCAE or PROMIS or FACIT or MDASI or questionnaire*or question or questions or sub-scale*))  23 (bespoke n3 (item or items or PRO or PROM or symptom or EORTC or PRO-CTCAE or PROMIS or FACIT or MDASI or questionnaire* or question or questions or sub-scale*))  24 (customi?ed n3 (item or items or PRO or PROM or symptom or EORTC or PRO-CTCAE or PROMIS or FACIT or MDASI or questionnaire* or question or questions or sub-scale*))  25 (item* n3 (item or items or PRO or PROM or symptom or EORTC or PRO-CTCAE or PROMIS or FACIT or MDASI or questionnaire* or question or questions or sub-scale*))  26 patient reported outcome measurement system  27 patient reported outcome librar*  28 searchable librar*  29 patient reported outcome item*  30 patient reported outcome item set  31 European organi?ation for research and treatment of cancer item library  32 MD Anderson symptom inventory  33 symptom librar*  34 Functional assessment of chronic illness therapy searchable library  35 Patient reported outcome* version of the common terminology criteria for adverse events  36 PRO item set  37 Patient reported outcome* item set  38 S19 OR S20 OR S21 OR S22 ORS23 OR S24 OR S25 OR S26 ORS27 OR S28 OR S29 OR S30 ORS31 OR S32 OR S33 OR S34 ORS35 OR S36 OR S37  39 S18 AND S38  40 Limiters - Publication Date: 20200101-20231231  41 S39 AND S40  42 Limiters - English Language; Exclude MEDLINE records; Human |

### Supplementary methods: results of the MMAT quality assessment

#### Quantitative RCT designs quality assessment

| Author/year | S1. Are there clear research questions? | S2. Do the collected data allow to address the research questions? | 2.1. Is randomization appropriately performed? | 2.2. Are the groups comparable at baseline? | 2.3. Are there complete outcome data? | 2.4. Are outcome assessors blinded to the intervention provided? | 2.5 Did the participants adhere to the assigned intervention? |
| --- | --- | --- | --- | --- | --- | --- | --- |
| Basch 2023 | Yes | Yes | Yes | Yes | Yes | No | Yes |
| Chung 2022 | Yes | Yes | Unclear | Yes | Yes | No | No |
| Madariaga 2022 | Yes | Yes | Unclear | Yes | Yes | Yes | Yes |
| Yeung 2022 | Yes | Yes | Yes | Yes | Yes | Unclear | Yes |
| Culakova 2022 | Yes | Yes | Yes | Yes | Yes | Yes | Yes |
| Saito 2025 | Yes | Yes | Yes | Yes | Yes | Yes | Yes |
| Ohri 2025 | Yes | Yes | Yes | Yes | Yes | No | Yes |
| Lv 2025 | Yes | Yes | Unclear | Yes | Yes | Yes | Yes |
| Hungria 2025 | Yes | Yes | Yes | Yes | Yes | No | Yes |
| Hummel 2025 | Yes | Yes | Yes | Yes | Yes | No | Yes |
| Compton 2025 | Yes | Yes | Yes | Yes | Yes | No | Yes |
| Bandos 2025 | Yes | Yes | Yes | Yes | Yes | Yes | Yes |
| Rugo 2024 | Yes | Yes | No | Yes | Yes | No | Yes |
| Oliveira 2024 | Yes | Yes | Yes | Yes | Yes | Yes | Yes |

#### Quantitative non-RCT designs quality assessment

| Author/year | S1. Are there clear research questions? | S2. Do the collected data allow to address the research questions? | 3.1. Are the participants representative of the target population? | 3.2. Are measurements appropriate regarding both the outcome and intervention (or exposure)? | 3.3. Are there complete outcome data? | 3.4. Are the confounders accounted for in the design and analysis? | 3.5. During the study period, is the intervention administered (or exposure occurred) as intended? |
| --- | --- | --- | --- | --- | --- | --- | --- |
| Adesoye 2023 | Yes | Yes | Yes | Yes | Yes | Yes | Yes |
| Anderson 2023 | Yes | Yes | Yes | Yes | Yes | Yes | Yes |
| Brunner 2023 | Yes | Yes | Yes | Yes | Unclear | Unclear | Unclear |
| Heino 2022 | Yes | Yes | Yes | Yes | Yes | Yes | Yes |
| Jacobs 2022 | Yes | Yes | Yes | Yes | Yes | Yes | Yes |
| Knoerl 2022 | Yes | Yes | Yes | Yes | Yes | Yes | Yes |
| Lapen 2022 | Yes | Yes | No | Yes | Yes | Unclear | Yes |
| L'Hotta 2023 | Yes | Yes | Yes | Yes | Yes | Yes | Yes |
| Ma 2023 | Yes | Yes | Yes | Unclear | No | Unclear | Yes |
| Martin 2022 | Yes | Yes | Yes | Yes | Yes | Unclear | Yes |
| Nelson 2023 | Yes | Yes | Yes | Yes | Yes | Yes | Yes |
| Nielsen 2022 | Yes | Yes | No | Yes | Yes | Yes | Unclear |
| Roziner 2023 | Yes | Yes | Yes | Yes | Yes | Yes | Yes |
| van der Weijst 2022 | Yes | Yes | Yes | Yes | Yes | Yes | Yes |
| deAlmeida 2023 | Yes | Yes | Yes | Yes | Unclear | Unclear | Yes |
| Kaveenuntachai 2025 | Yes | Yes | Yes | Yes | Unclear | Unclear | Yes |
| Carter 2025 | Yes | Yes | Yes | Yes | No | Yes | Yes |
| Schuler 2025 | Yes | Yes | Unclear | No | No | Unclear | Yes |
| Akmansu 2025 | Yes | Yes | Yes | Yes | Yes | Unclear | Yes |
| Ya-Jung 2024 | Yes | Yes | Yes | Yes | Yes | Unclear | Yes |
| vandeWal 2024 | Yes | Yes | Yes | Yes | Unclear | Unclear | Yes |
| Ruddy 2024 | Yes | Yes | No | Yes | No | Unclear | Yes |
| Lin 2024 | Yes | Yes | Yes | Yes | Unclear | Yes | Yes |
| Kurosawa 2024 | Yes | Yes | Yes | Yes | Unclear | Unclear | Yes |

#### Quantitative descriptive designs quality assessment

| Author/year | S1. Are there clear research questions? | S2. Do the collected data allow to address the research questions? | 4.1. Is the sampling strategy relevant to address the research question? | 4.2. Is the sample representative of the target population? | 4.3. Are the measurements appropriate? | 4.4. Is the risk of nonresponse bias low? | 4.5. Is the statistical analysis appropriate to answer the research question? |
| --- | --- | --- | --- | --- | --- | --- | --- |
| Dierickx 2022 | Yes | Yes | Yes | Yes | Yes | Yes | Yes |
| Hsu 2022 | Yes | Yes | Yes | Yes | Yes | Unclear | Yes |
| Jakob 2022 | Yes | Yes | Yes | Yes | Yes | No | Yes |
| JooMiPark 2023 | Yes | Yes | Yes | Yes | Yes | Unclear | Yes |
| Patel 2023 | Yes | Yes | Yes | Yes | Yes | No | Yes |
| Ruan 2023 | Yes | Yes | Yes | Yes | Yes | Unclear | Yes |
| Thorpe 2022 | Yes | Yes | Yes | Yes | Yes | Unclear | Yes |
| Walker 2021 | Yes | Yes | Yes | Yes | Yes | Yes | Yes |
| Wang 2021 | Yes | Yes | Yes | Yes | Yes | Yes | Yes |
| Whisenant 2022 | Yes | Yes | Yes | Yes | Yes | Unclear | Yes |
| Withycombe 2022 | Yes | Yes | Yes | Yes | Yes | Yes | Yes |
| Wujcik 2022 | Yes | Yes | Yes | Yes | Yes | Yes | Yes |
| Balachandran 2024 | Yes | Yes | Yes | Yes | Yes | Yes | Yes |
| Bjornholt 2025 | Yes | Yes | Yes | Yes | Yes | Yes | Yes |
| Chevallay 2025 | Yes | Yes | Yes | Yes | Yes | Yes | Yes |
| Conti 2025 | Yes | Yes | Yes | Yes | Yes | Unclear | Yes |
| David 2024 | Yes | Yes | Yes | Yes | Yes | Yes | Yes |
| Gabbard 2024 | Yes | Yes | Yes | Yes | Yes | Yes | Yes |
| Haishan 2024 | Yes | Yes | Yes | Yes | Yes | Unclear | Yes |
| Han 2025 | Yes | Yes | Yes | Yes | Yes | Unclear | Yes |
| Horan 2024 | Yes | Yes | Yes | Yes | Yes | Yes | Yes |
| Kamimura 2024 | Yes | Yes | Yes | Yes | Yes | Unclear | Yes |
| Lau 2025 | Yes | Yes | Yes | Yes | Yes | Yes | Yes |
| McDowell 2025 | Yes | Yes | Yes | Yes | Yes | Yes | Yes |
| Mittal 2025 | Yes | Yes | Yes | Yes | Yes | Yes | Yes |
| Muaddi 2025 | Yes | Yes | Yes | Yes | Yes | No | Yes |
| Nash 2024 | Yes | Yes | Yes | Yes | Yes | No | Yes |
| Nugent 2024 | Yes | Yes | Yes | Yes | Yes | Unclear | Yes |
| Nyrop 2025 | Yes | Yes | Yes | Yes | Yes | Yes | Yes |
| Patel 2024 | Yes | Yes | Yes | Yes | Yes | Unclear | Yes |
| Sakaguchi 2023 | Yes | Yes | Yes | Yes | Yes | Yes | Yes |
| Shawahna 2023 | Yes | Yes | Yes | Yes | Yes | No | Yes |
| Smith 2024 | Yes | Yes | Yes | Yes | Yes | Unclear | Yes |

#### Mixed method designs quality assessment

| Author/year | S1. Are there clear research questions? | S2. Do the collected data allow to address the research questions? | 5.1. Is there an adequate rationale for using a mixed methods design to address the research question? | 5.2. Are the different components of the study effectively integrated to answer the research question? | 5.3. Are the outputs of the integration of qualitative and quantitative components adequately interpreted? | 5.4. Are divergences and inconsistencies between quantitative and qualitative results adequately addressed? | 5.5. Do the different components of the study adhere to the quality criteria of each tradition of the methods involved? |
| --- | --- | --- | --- | --- | --- | --- | --- |
| denHollander 2022 | Yes | Yes | Yes | Yes | Yes | Yes | Yes |
| Nordhausen 2022 | Yes | Yes | Yes | Yes | Yes | No | Yes |
| Uneno 2024 | Yes | Yes | Yes | Yes | Yes | Yes | Unclear |
| Jackson-Carroll 2024 | Yes | Yes | Yes | Yes | Yes | Yes | Yes |
| Gomaa 2023 | Yes | Yes | Yes | Yes | Yes | Yes | Unclear |
| Fridriksdottir 2023 | Yes | Yes | Yes | Yes | Yes | Yes | Yes |

#### Qualitative designs quality assessment

| Author/year | S1. Are there clear research questions? | S2. Do the collected data allow to address the research questions? | 5.1. Is there an adequate rationale for using a mixed methods design to address the research question? | 5.2. Are the different components of the study effectively integrated to answer the research question? | 5.3. Are the outputs of the integration of qualitative and quantitative components adequately interpreted? | 5.4. Are divergences and inconsistencies between quantitative and qualitative results adequately addressed? | 5.5. Do the different components of the study adhere to the quality criteria of each tradition of the methods involved? |
| --- | --- | --- | --- | --- | --- | --- | --- |
| O'Leary 2024 | Yes | Yes | Yes | Yes | Yes | Yes | Yes |
